# Meeting Abstracts of the first meeting of the American Society for Intercellular Communication 2021

**DOI:** 10.20517/evcna.2022.10

**Published:** 2022-07-19

**Authors:** Gurudutt Pendyala, Ashley E. Russell, Shilpa Buch, Susmita Sil, Emeli Chatterjee, Bojan Losic, Alissa M. Weaver, Tsuneya Ikezu, Randy Schekman, Xiaoli Yu

*1st meeting of the American Society for Intercellular Communication (ASIC), October 21-23, 2021 [Table 1].

**Table 1:** Table of Content

**No.**	**Abstract Title**	**Authors**
1	R(EV)ealing Sex Differences with Nicotine Addiction	Gurudutt Pendyala
2	Cortisol’s Effects on Iron Transport Proteins and EV Release in Placental Cells	Sophie C. Anderlind, Ashley E. Russell
3	The Effects of Endoplasmic Reticulum Stress on Oligodendrocyte Derived EVs	Madison T. Jones, Samantha W. Manioci, Ashley E. Russell
4	HIV-1 Tat Primes and Activates Microglial NLRP3 Inflammasome Leading to Synaptodendritic Injury in Neurons via Exosomes	Shilpa Buch, Ernest T. Chivero, Susmita Sil, Seema Singh, Abiola Oladapo, Muthukumar Kannan
5	Astrocyte-Derived Extracellular Vesicles in Morphine Induced Synaptodegeneration	Susmita Sil, Shilpa Buch
6	An Organ-On-Chip Model to Characterize Extracellular Vesicles as Functional Biomarkers in Cardiorenal Syndrome	Emeli Chatterjee, Ville Kujala, Rodos Rodosthenous, Parul Sahu, Michail Spanos, Katia Karalis, Saumya Das
7	Unannotated Small RNA Clusters Associated with Circulating Extracellular Vesicles Detect Early Stage Liver Cancer	Johann von Felden, Teresa Garcia-Lezana, Navneet Dogra, Edgar Gonzalez-Kozlova, Mehmet Eren Ahsen, Amanda Craig, Stacey Gifford, Benjamin Wunsch, Joshua T Smith, Sungcheol Kim, Jennifer E L Diaz, Xintong Chen, Ismail Labgaa, Philipp Haber, Reena Olsen, Dan Han, Paula Restrepo, Delia D’Avola, Gabriela Hernandez-Meza, Kimaada Allette, Robert Sebra, Behnam Saberi, Parissa Tabrizian, Amon Asgharpour, Douglas Dieterich, Josep Llovet, Carlos Cordon-Cardo, Ash Tewari, Myron Schwartz, Gustavo Stolovitzky, Bojan Losic, Augusto Villanueva
8	Biogenesis of RNA-Containing Extracellular Vesicles at Endoplasmic Reticulum Membrane Contact Sites	Bahnisikha Barman, Jie Ping, Evan Krystofiak, Ryan Allen, Nripesh Prasad, Kasey Vickers, James G. Patton, Qi Liu, Alissa M. Weaver
9	Cell Type-Specific EV Define Disease-Related Protein Networks Associated with Astrocyte Activation in Alzheimer’s Disease	Tsuneya Ikezu
10	Selective Protein Sorting into Exosomes: A Role in Cell Differentiation and a Possible Tool in Genome Editing	Randy Schekman, Lu Song, Congyan Zhang
11	Characterizing IgG Antibodies in Plasma and Extracellular Vesicles of Patients with Glioblastoma and Meningioma	Daniel Lovasz, Zoë Zizzo, Wenbo Zhou, Qianbin He, Arin N Graner, Kevin O Lillehei, D Ryan Ormond, A Samy Youssef, Robert G Kowalski, Michael W Graner, Xiaoli Yu

## 1. R(EV)ealing sex differences with nicotine addiction

Gurudutt Pendyala

Department of Anesthesiology, University of Nebraska Medical Center, Omaha, NE 68198, USA.

Smoking remains a significant health and economic concern in the United States. Furthermore, the emerging pattern of nicotine intake between sexes further adds a layer of complexity. Nicotine is a potent psychostimulant with a high addiction liability that can significantly alter brain function. However, the neurobiological mechanisms underlying nicotine’s impact on brain function and behavior remain unclear. Elucidation of these mechanisms is of high clinical importance and may lead to improved therapeutics for smoking cessation. To fill in this critical knowledge gap, our recent study focused on identifying sex-specific brain-derived extracellular vesicles (BDEV) signatures in male and female rats post nicotine self-administration. Interestingly, females post nicotine self-administration showed larger BDEV sizes and impaired EV biogenesis than males. Next, using quantitative mass spectrometry-based proteomics, we identified BDEV signatures, including distinct molecular pathways, impact among males and females. We now are employing a novel technology, Single EV analyses with multiplexing (SEAM), to validate these identified sex-specific BDEV markers in the blood plasma from preclinical and clinical samples and further extend our studies into a model of relapse.

## 2. Cortisol’s effects on iron transport proteins and EV release in placental cells

Sophie C. Anderlind, Ashley E. Russell

Department of Biology, School of Science, Pennsylvania State University, Erie, PA 16563, USA.

Although the placenta’s significance is commonly unrecognized, a healthy pregnancy relies heavily on the placenta’s role to function for the fetus’ lungs, heart, kidney, and other vital underdeveloped organs. Extracellular vesicles (EVs) are important intracellular communicators essential to an optimally functioning placenta because they contain bioactive cargo released during both normal and pathological cellular activities. Previous work has shown that EVs are involved in maternal-to-fetal trafficking of important proteins, including iron transport proteins. While iron is an essential nutrient that transports oxygen for fetal development, a surplus or inadequate iron level can lead to negative pregnancy outcomes. The regulation of iron transport proteins is mediated by several factors, and previous research has shown that stress hormones, such as cortisol, have been observed to alter EV release. Three key iron transporting proteins in the trophoblast placental layer that facilitate nutrient absorption between the maternal and fetal bloodstream are Transferrin receptor 1 (TFR1), Ferroportin-1, and DMT-1. In the current study, we are investigating the effects of hydrocortisone (cortisol) on the cellular expression of these iron transport proteins and determining whether they are present in EVs. We are exposing a placental trophoblast cell line, BeWo, to physiologically relevant concentrations of hydrocortisone and assessing the expression of these iron transport proteins via western blot. Additionally, we are using size exclusion chromatography to separate EVs from the conditioned cell culture media in order to examine changes in their protein composition after hydrocortisone exposure. Our results will demonstrate the effects of cortisol exposure on iron transport proteins, which may influence how iron is delivered during fetal development.

## 3. The effects of endoplasmic reticulum stress on oligodendrocyte derived EVs

Madison T. Jones, Samantha W. Manioci, Ashley E. Russell

Department of Biology, School of Science, Pennsylvania State University, Erie, PA 16563, USA.

Oligodendrocytes are a type of glial cell found in the central nervous system, which form myelin sheaths that insulate neuronal axons. Myelin sheaths contain various proteins and lipids that are necessary for their formation. The proteins and membranes are synthesized, folded, and transported in the cell’s endoplasmic reticulum (ER). Disruption of the ER’s protein synthesis mechanisms can cause ER stress due to the accumulation of misfolded and unfolded proteins. Adequate protein production is crucial for oligodendrocytes to form their myelin sheaths properly; as such, these cells are particularly susceptible to the negative effects of ER stress. Previous work has demonstrated that ER stress induces EV release in some cell types. In the current study, we are determining how ER stress affects EV release and protein composition in oligodendrocytes. To achieve this, we are culturing human oligodendroglioma (HOG) cells and exposing them to tunicamycin to induce ER stress. To confirm the successful induction of ER stress, we are assessing the expression of three proteins that become activated in response to ER stress: inositol-requiring enzyme 1 α (IRE1α), protein kinase R (PKR) endoplasmic reticulum kinase (PERK), and activating transcription factor 6 (ATF6). To determine how ER stress affects EV release and composition, we separate EVs from the conditioned cell culture media of control and tunicamycin treated cells by size exclusion chromatography and examining changes in their protein composition via western blot. We also exposinge naïve oligodendrocytes to EVs from ER stressed oligodendrocytes to determine the role of ER stress-induced oligodendrocyte-derived EVs in ER-stress mediated pathology. These results can provide insight into our understanding of the pathophysiology of neurodegenerative diseases associated with demyelination, including amyotrophic lateral sclerosis (ALS) and multiple sclerosis (MS).

## 4. HIV-1 tat primes and activates microglial NLRP3 inflammasome leading to synaptodendritic injury in neurons via exosomes

Shilpa Buch, Ernest T. Chivero, Susmita Sil, Seema Singh, Abiola Oladapo, Muthukumar Kannan

Department of Pharmacology and Experimental Neuroscience, University of Nebraska Medical Center, Omaha, Nebraska 68198, USA.

Neuroinflammation associated with HIV-1 infection affects 50% of HIV-infected individuals. NLR family pyrin domain containing 3 (NLRP3) inflammasome has been implicated in HIV-induced microglial activation; however, the mechanism(s) underlying this remain elusive. Since HIV-1 Transactivator of Transcription (Tat) protein persists despite antiretroviral therapy and, as a result, activates the NF-kB pathway, we hypothesized that Tat protein could prime the NLRP3 inflammasome, which, in turn, could be released by the EVs, ultimately leading to synaptodendritic injury in neurons. As expected, there was an induction of NLRP3 expression in microglia exposed to Tat compared with cells not exposed to Tat. Tat exposure of microglia also time-dependently increased the expression levels of mature caspase-1 and IL-1β as well as IL-1β secretion. These *in vitro* findings were validated in archival postmortem brain tissues from Simian Immunodeficiency Virus (SIV)-infected and uninfected rhesus macaques. Further validation of NLRP3 priming *in vivo* involved administration of lipopolysaccharide (LPS) to HIV transgenic (Tg) rats, followed by assessment of IL-1β mRNA expression and inflammasome activation (ASC oligomers and mature IL-1). Intriguingly, LPS potentiated upregulation of IL- 1β mRNA and inflammasome activation in HIV-Tg rats compared with the wild-type controls. Interestingly, we found an inverse relationship in the expression of NLRP3 and its negative regulator, miR-223, suggesting a miR-223-mediated mechanism for Tat-induced NLRP3 priming. Furthermore, blockade of NLRP3 resulted in decreased IL-1β secretion. Taken together, our findings suggest that microglial NLPR3 can be released in Tat-EVs, resulting in synaptodendritic injury in neurons. Currently, we are investigating the protective role of silencing NLRP3 in ameliorating synaptic degeneration. In summary, our findings suggest a novel role of Tat in priming and activating the NLRP3 inflammasome, which can be carried via the EVs to recipient neurons, leading to neuronal injury. NLRP3 can thus be envisioned as a therapeutic target for ameliorating Tat-mediated neuroinflammation as well as EV-mediated synaptic neurodegeneration.

Acknowledgment: This work is supported by RO1DA044586, DA040397, DA043138, DA047156, DA050545 (S. Buch).

## 5. Astrocyte-derived extracellular vesicles in morphine induced synaptodegeneration

Susmita Sil, Shilpa Buch

Department of Pharmacology and Experimental Neuroscience, University of Nebraska Medical Center, Omaha, NE, Nebraska 68198, USA.

A study from our group has revealed the role of morphine-induced neuronal autophagy in mediating synaptodendritic injury. The role of other CNS cell types, such as the astrocytes, in contributing to morphine-mediated neuronal injury, however, remains an enigma. Several studies have identified interactive crosstalk and shared molecular machinery between extracellular vesicle biogenesis & the autophagy pathway. The current study was undertaken to understand whether morphine-mediated dysregulation of autophagy in astrocytes could also mediate neuronal dysfunction via the EVs. Based on our initial findings that morphine-initiated autophagy in astrocytes while blocking the autophagy flux, we sought to understand the link between dysregulated autophagy and EV biogenesis. Our results showed that morphine exposure induced expression of NMDA-NR1 subunit in the human astrocytes, which, in turn, led to the initiation of autophagy and formation of autophagosome, and a concomitant block in the autophagic flux. Intriguingly, morphine-mediated block in autophagic flux was accompanied by increased release of astrocyte-derived EVs containing the autophagic proteins. Next, we assessed whether the neurons could take up morphine-stimulated ADEVs and if so, the extent of synaptic impairment, if any. For this, rat hippocampal neurons were exposed to morphine-ADEVs carrying the autophagic proteins and assessed for spine morphology. Exposure of neurons to the morphine-ADEVs resulted in an increase in immature dendritic spines, a reduction in excitatory synapse densities, with a concomitant increase in numbers of inhibitory synapses, leading to synaptic dysfunction. Silencing of astrocytic NMDA-NR1 not only reduced the release of ADEVs and their autophagy cargoes, but also ameliorated synaptodegeneration. This study underscores the role of autophagy cargoes in morphine-ADEVs mediated neuronal injury and synaptic alterations. Understanding how morphine hijacks the autophagy machinery to regulate EV release via the astrocytic NMDA-NR1 and how this phenomenon is involved with neurodegeneration is a novel concept, which can set the groundwork for future development of therapeutics for opiate addicts.

Acknowledgment: This work is supported by RO1DA044586, DA040397, DA043138, DA047156, DA050545 (S. Buch).

## 6. An organ-on-chip model to characterize extracellular vesicles as functional biomarkers in cardiorenal syndrome

Emeli Chatterjee^1^, Ville Kujala^2^, Rodos Rodosthenous^1^, Parul Sahu^1^, Michail Spanos^1^, Katia Karalis^2^, Saumya Das^1^

^1^Cardiovascular Division of the Massachusetts General Hospital and Harvard Medical School, Boston, MA 02114, USA.

^2^Emulate, Inc, 27 Drydock Ave, Boston, MA 02210, USA.

Extracellular vesicles (EVs) and their cargo have diverse roles in mediating intercellular signaling and can thus serve as potential functional circulating biomarkers. Type 1 cardiorenal syndrome (CRS) is characterized by the development of acute kidney injury in the setting of acute decompensated heart failure (ADHF) and can complicate the clinical care of HF patients. However, there is a paucity of biomarkers and mechanistic understanding of CRS. The emergence of micro-fluidic organ-on-chip platforms comprising human tissues that can faithfully recapitulate key aspects of human physiology opens the door to studying the functional role of EVs in type I CRS. In this study, we used the Kidney Proximal TubuleChip model (Emulate) to elucidate the possible role of EVs in CRS.

EVs were isolated from patients with heart failure with preserved ejection fraction (HFpEF) with or without CRS and healthy controls, followed by treatment with RNAseA and dosed on Kidney Proximal Tubule-Chips a single bolus in the vascular channel and administered for 72 hours. Each chip includes epithelial cells in the top channel and endothelial cells in the bottom channel. EV uptake was observed using fluorescent tagging of EVs prior to perfusion in the chamber. Following the treatment, RNA was isolated from both top and bottom channels of each chip, expression of kidney injury markers like Neutrophil gelatinase-associated lipocalin (NGAL) and Interleukin-18 (IL-18) were measured by qPCR. 

Isolated EVs were characterized following MISEV guidelines. Dil-stained EVs from healthy control subjects were visualized after a three-day perfusion period using fluorescence microscopy, confirming successful EVs uptake on tissue chip. After treatment with EVs, differential expression levels of mRNA of NGAL and IL-18 were observed in human glomerular endothelial cells and proximal tubule epithelial cells. NGAL and IL-18were significantly increased in both endothelial and epithelial cells in response to incubation with EVs from HF patients. The increase was higher in HF patients with CRS than those without CRS. 

Our tissue on-chip study bridges the gap between *in vitro* and *in vivo* models offering new approaches to identify the role of plasma EVs as potential biomarker for CRS.

## 7. Unannotated small RNA clusters associated with circulating extracellular vesicles detect early stage liver cancer

Johann von Felden^1,2^, Teresa Garcia-Lezana^2^, Navneet Dogra^3,4^, Edgar Gonzalez-Kozlova^3^, Mehmet Eren Ahsen^3^, Amanda Craig^5^, Stacey Gifford^4^, Benjamin Wunsch^4^, Joshua T Smith^4^, Sungcheol Kim^4^, Jennifer E L Diaz^3^, Xintong Chen^6^, Ismail Labgaa^6,7^, Philipp Haber^5^, Reena Olsen^8^, Dan Han^8^, Paula Restrepo^3^, Delia D’Avola^5^, Gabriela Hernandez-Meza^6^, Kimaada Allette^3^, Robert Sebra^3^, Behnam Saberi^9^, Parissa Tabrizian^10^, Amon Asgharpour^11^, Douglas Dieterich^12^, Josep Llovet^11^, Carlos Cordon-Cardo^3,8^, Ash Tewari^13^, Myron Schwartz^14^, Gustavo Stolovitzky^3,4^, Bojan Losic^3,15^, Augusto Villanueva^6^

^1^Department of Internal Medicine, Universitätsklinikum Hamburg-Eppendorf, Hamburg 20246, Germany.

^2^Division of Liver Diseases, Department of Medicine, Tisch Cancer Institute, Icahn School of Medicine at Mount Sinai, New York, NY 10029-5674, USA.

^3^Department of Genetics, Icahn School of Medicine at Mount Sinai, New York, NY 10029-5674, USA.

^4^IBM Thomas J Watson Research Center, Yorktown Heights, New York, NY 10598, USA.

^5^Tisch Cancer Institute, Icahn School of Medicine at Mount Sinai, New York, NY 10029-5674, USA.

^6^Department of Medicine, Icahn School of Medicine at Mount Sinai, New York, NY 10029-5674, USA.

^7^Department of Visceral Surgery, Lausanne University Hospital, Lausanne 1011, Switzerland.

^8^Department of Pathology, Icahn School of Medicine at Mount Sinai, New York, NY 10029-5674, USA.

^9^Department of Medicine-Liver Diseases, Tisch Cancer Institute, Icahn School of Medicine at Mount Sinai, New York, NY 10029-5674, USA. 

^10^Recanati/Miller Transplantation Institute, Icahn School of Medicine at Mount Sinai, New York, NY 10029, USA.

^11^Division of Liver Diseases, Icahn School of Medicine at Mount Sinai, New York, NY 10029-5674, USA.

^12^Division of Liver Diseases, Mount Sinai School of Medicine, New York, NY 10029-5674, USA.

^13^Department of Urology, Icahn School of Medicine at Mount Sinai, New York, NY 10029-5674, USA.

^14^Department of Surgery, Mount Sinai School of Medicine, New York, NY 10029-5674, USA.

^15^Guardant Health, Redwood City, California, CA 94064, USA.

Objective Surveillance tools for early cancer detection are suboptimal, including hepatocellular carcinoma (HCC), and biomarkers are urgently needed. Extracellular vesicles have gained increasing scientific interest due to their involvement in tumor initiation and metastasis; however, most extracellular RNA (exRNA) blood-based biomarker studies are limited to annotated genomic regions. EVs were isolated with ultracentrifugation and nanoDLD and quality was assessed by electron microscopy, immunoblotting, nanoparticle tracking and deconvolution analysis. Genome-wide sequencing of the largely unexplored small exRNA landscape, including unannotated transcripts, identified and reproducibly quantified small RNA clusters (smRCs). Their key genomic features were delineated across biospecimens and EV isolation techniques in prostate cancer and HCC. Three independent exRNA cancer datasets with a total of 479 samples from 375 patients, including longitudinal samples, were used for this study.

ExRNA smRCs were dominated by uncharacterized, unannotated small RNA with a consensus sequence of 20 bp. An unannotated 3-smRC signature was significantly overexpressed in plasma exRNA of patients with HCC (*P* < 0.01, *n* = 157). An independent validation in a phase 2 biomarker case-control study revealed 86% sensitivity and 91% specificity for the detection of early HCC from controls at risk (*n* = 209) [area under the receiver operating curve (AUC): 0.87]. The 3-smRC signature was independent of alphafetoprotein (*P* < 0.0001) and a composite model yielded an increased AUC of 0.93.

These findings directly lead to the prospect of a minimally invasive, blood-only, operator-independent clinical tool for HCC surveillance, thus highlighting the potential of unannotated smRCs for biomarker research in cancer.

## 8. Biogenesis of RNA-containing extracellular vesicles at endoplasmic reticulum membrane contact sites

Bahnisikha Barman^1^, Jie Ping^2^, Evan Krystofiak^3^, Ryan Allen^4^, Nripesh Prasad^5^, Kasey Vickers^4^, James G. Patton^6^, Qi Liu^2^, Alissa M. Weaver^1,7^

^1^Department of Cell and Developmental Biology, Vanderbilt University School of Medicine, Nashville, TN 37232, USA.

^2^Department of Biostatistics, Vanderbilt University Medical Center, Nashville, TN 37232, USA.

^3^Cell Imaging Shared Resource, Vanderbilt University, Nashville, TN 37232, USA.

^4^Department of Medicine, Vanderbilt University Medical Center, Nashville, TN 37232, USA.

^5^Hudson Alpha Institute for Biotechnology, Huntsville, AL 35806-2908, USA.

^6^Department of Biological Sciences, Vanderbilt University, Nashville, TN 37232, USA.

^7^Department of Pathology, Microbiology and Immunology, Vanderbilt University, Nashville, TN 37232, USA.

Extracellular RNAs carried by extracellular vesicles can affect gene expression, function, and phenotypes of recipient cells. While a number of RNA binding proteins (RBPs) are known to carry RNAs into extracellular vesicles, where and how in the cell this occurs is unclear. Here, we identify VAP-A positioned endoplasmic reticulum membrane contact sites (ER MCS) as key locations for the biogenesis of RNA-containing EVs.

We used RNA-sequencing, lipidomic, confocal and transmission electron microscopy, tumor xenograft and various biochemical techniques to analyze EV biogenesis and cargo content in colon cancer cell lines molecularly engineered for molecules that control ER MCS.

RNA-Seq analysis revealed a number of small RNAs that are altered in VAP-A KD small and large EVs compared to control cells. Density gradient fractionation revealed that VAP-A regulates a select subpopulation of small EVs enriched with RNA and RBPs. Furthermore, this VAP-A-controlled small EV population is critical for transferring miR-100 to recipient cells and the growth of xenograft mouse tumors. Analysis of small and large EVs for lipid content revealed that VAP-A controls the levels of ceramide and cholesterol, two lipids involved in EV biogenesis. Furthermore, KD of the VAP-A binding ceramide and cholesterol transporters CERT and ORP1L led to similar defects in EV biogenesis.

We uncovered a novel pathway of EV biogenesis that takes place at ER MCS. These data suggest a model in which lipid transfer at ER MCS drives the biogenesis of a select subpopulation of EVs containing RNA-RBP complexes. Beyond improving our understanding of EV biogenesis, we anticipate that these findings may be helpful in future engineering of therapeutic EVs as well as exploring the functions of RNA-containing EVs.

Funding: Funding was provided by NIH grants U19CA179514 and P01CA229123; Vanderbilt CTSA grant UL1 RR024975 and UL1 TR002243.

## 9. Cell type-specific EV define disease-related protein networks associated with astrocyte activation in Alzheimer’s disease

Tsuneya Ikezu

Department of Neuroscience, Mayo Clinic Florida, Jacksonville, FL 32224, USA.

Extracellular vesicles (EVs) are known to transfer pathogenic molecules in neurodegenerative diseases and are involved in disease progression. Here we investigated the proteomic profile of EVs isolated from human induced pluripotent stem cell (hiPSC)-derived neural cells. Novel cell type-specific EV protein markers are identified for excitatory neurons (ATP1A3, NCAM1), astrocytes (LRP1, ITGA6), microglia-like cells (ITGAM, CD300A) and oligodendrocyte-like cells (LAMP2, FTH1), as well as 16 pan-EV marker candidates including integrins and annexins. To further demonstrate how cell type-specific EVs may interplay in Alzheimer’s disease (AD), we performed protein co-expression network analysis with cell type assessment of proteomes of brain-derived EVs from the control, mild cognitive impairment, and AD cases. A protein module enriched in astrocyte-specific EV markers was found most significantly associated with AD pathology and cognitive impairment, suggesting the important role it may play in AD progression. Finally, we confirmed that the hub protein from this module, integrin-β1 (ITGB1), was elevated in AD astrocyte-derived EVs purified from total brain-derived EVs and associated with brain Aβ42 and tau load in independent cohorts. Thus, our study provides a featured framework and rich resource for analyses of EV functions on neurodegenerative diseases in a cell type-specific manner.

## 10. Selective protein sorting into exosomes: a role in cell differentiation and a possible tool in genome editing

Randy Schekman, Lu Song, Congyan Zhang

Department of Molecular and Cell Biology, Howard Hughes Medical Institute, University of California, Berkeley, CA 94720-3200, USA.

Extracellular vesicles (EVs) are thought to mediate the transfer of cytoplasmic proteins and RNA between cells to inform the processes of differentiation, cell motility and possibly malignant transformation. We have found that undifferentiated or embryonic stem cells (ESCs), which are induced to differentiate into neural progenitor cells, secrete EVs that selectively capture proteins implicated in G1/S cell cycle progression. Further, we have found that these EVs promote the differentiation of ESCs to a neural progenitor fate and that cyclin D1 plays a rate-limiting role in that EV-mediated progression.

A challenge in genome editing *in vivo* is to devise an efficient means of delivering editing functions, preferably by a vehicle that evades immune detection. We sought a means to deliver Cas9 and a gRNA enclosed within exosomes, a subclass of EVs, as a vehicle for efficient and targeted gene editing. Cas9 was expressed in a donor cell tethered noncovalently to an integral membrane protein, CD63, enriched in exosomes. Exosomes highly enriched in Cas9 and a gRNA were isolated by buoyant density sedimentation. Isolated exosomes were incubated with reporter cells containing an integrated copy of N-luciferase behind a site that, when edited, would allow the luciferase expression. In a control experiment, expression of the Cas9/gRNA constructs directly in the reporter cell elicited a 60-70 fold increase in luciferase expression. Exosomes containing a similar level of Cas9 elicited no more than a 50% increase above the background of luciferase. The same was true of conditioned medium containing Cas9-exosomes and even of donor and acceptor cells incubated together, separated by a vesicle-permeable membrane in a transwell chamber. Thus, for these EVs, the functional uptake to promote gene expression was not observed as we found for those isolated from differentiating neurons. In contrast, donor and acceptor cells cocultured to near confluence showed a 60-fold increase in luciferase expression. Transfer of Cas9 appears to be mediated by open-end membrane tubular connections, likely dependent on membrane fusion at the junction point between a tubule from one cell and the target. Molecular dissection of the requirements for this transfer may permit the development of an efficient means for targeted delivery of Cas9/gRNA.

## 11. Characterizing IgG antibodies in plasma and extracellular vesicles of patients with glioblastoma and meningioma

Daniel Lovasz^#^, Zoë Zizzo^#^, Wenbo Zhou, Qianbin He, Arin N Graner, Kevin O Lillehei, D Ryan Ormond, A Samy Youssef, Robert G Kowalski, Michael W Graner, Xiaoli Yu

Department of Neurosurgery, University of Colorado Anschutz Medical Campus, Aurora, CO 80045, USA.

^#^Authors contributed equally.

Glioblastoma (GBM) is the most common malignant primary brain tumor in humans. Meningioma (MMA) is a generally benign brain tumor formed from the meningeal layers of the brain; 10%-15% of these tumors become malignant. Previous findings suggest that tumor antibodies have decreased function from subtle proteolytic cleavage. Therefore, we hypothesized that immunoglobulin G (IgG) antibodies in the plasma of brain tumor patients are abnormal and may play a significant role in tumor pathogenesis. Using multiple immunoassays, we characterized IgG antibodies in plasma and plasma extracellular vesicles (EVs) from patients with GBM (*n* = 82), MMA(*n* = 83), and controls (non-tumor CNS disorders and healthy donors, *n* = 50). By capturing ELISA, we found that significantly higher levels of Fc heavy chain IgG antibodies and IgG1 subclass are present in GBM plasma compared to MMA (*P* = 0.0002 and *P* = 0.0003, respectively). Similarly, EVs purified from GBM plasma contained higher IgG Fc heavy chain levels than that of MMA. In addition, immunohistochemistry demonstrated the presence of IgG antibodies in GBM tumors tissues, concentrating around the periphery of the tumor. Importantly, we demonstrated that the IgG antibodies in both GBM plasma and EVs produce complement-dependent cytotoxicity on a neuroblastoma cell line SH-SY5Y used as a neuronal surrogate. The higher IgG levels in the plasma and EVs in GBM patients and the high cell killing capacity suggest that GBM IgG antibodies may play an important role in tumor pathogenesis.

